# Patterns of damage observed on dimercaptosuccinic acid kidney scans and future risk of urinary tract infections or reduced kidney function

**DOI:** 10.1007/s00467-025-06779-1

**Published:** 2025-04-28

**Authors:** Nadin Kanaan, Shiri Cooper, Daniel Landau, Zvi Bar Sever, Orly Haskin

**Affiliations:** 1 Department of Pediatrics B, Schneider Children’s Medical Center of Israel, Petah-Tikva, Israel; 2 Institute of Nephrology, Schneider Children’s Medical Center of Israel, Petah-Tikva, Israel; 3 Faculty of Medicine, Tel Aviv University, Tel Aviv, Israel; 4 Department of Nuclear Medicine, Schneider Children’s Medical Center of Israel, Petah-Tikva, Israel

**Keywords:** Kidney scars, UTI, DMSA scan, Pediatrics

## Abstract

**Background:**

^99m^Tc-Dimercaptosuccinic acid (DMSA) scan is highly accurate for assessing functional imaging of the kidney parenchyma. Kidney damage observed on DMSA scan is associated with future development of chronic kidney disease. This study aims to differentiate between different patterns of damage observed on DMSA scan and determine their predictive clinical value.

**Methods:**

We reviewed first-in-life DMSA scans performed ≥ 4 months post febrile urinary tract infection (UTI) or for suspected congenital kidney abnormalities, in a single referral center, from November 2007 to February 2011. DMSA uptake patterns were classified as normal; peripheral focal defects; diffuse inhomogeneity in tracer distribution within kidney parenchyma; and the combination of both patterns. Subsequent UTIs and estimated glomerular filtration rate (eGFR) were recorded at last follow-up.

**Results:**

One hundred five patients met inclusion criteria, and 57 (54%) were females. Median (IQR) age at scan was 2 (1.3, 5.1) years. Fourteen patients (13.3%) had focal defects, 29 (27.6%) had diffuse inhomogeneity and 9 (8.6%) had diffuse inhomogeneity with focal defects. After a mean follow-up period of 9.6 ± 3.3 years (available for 99 children), 29 (29%) patients experienced recurrent UTIs [median (IQR) episodes: 2 (1, 5)]. UTI tendency differed between groups (focal defects: 71.4%; diffuse inhomogeneity with focal defect: 44.4%; diffuse inhomogeneity only: 22.2%; normal scan: 18.3% *p* < 0.001). On multivariate analysis only the presence of focal defects predicted recurrent UTIs [OR (95%CI): 3.89 (1.2, 12.6), *p* = 0.024]. The percentage of patients with an eGFR < 75 ml/min/1.73 m^2^, was highest in patients with diffuse inhomogeneity with focal defects compared to patients with normal scans, focal defects only or diffuse inhomogeneity only (22% vs. 2%, 0% and 3.7% respectively, *p* = 0.032).

**Conclusions:**

Focal defects on DMSA scan, likely representing post pyelonephritis scars, are a strong predictor of recurrent UTIs. Patients with diffuse inhomogeneity with focal defects on scan have the highest risk of reduced eGFR during follow-up.

**Graphical abstract:**

**Figure:**
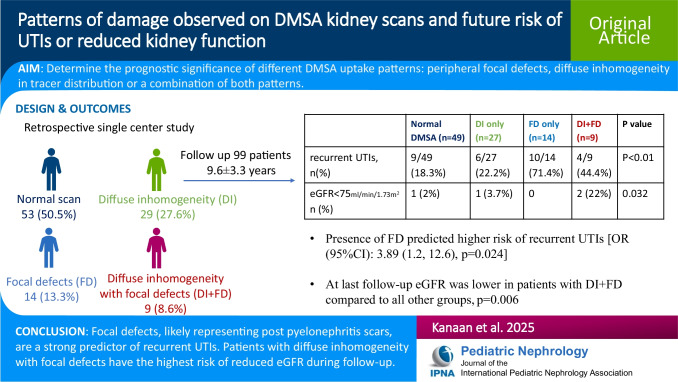
A higher resolution version of the Graphical abstract is available as Supplementary information

**Supplementary Information:**

The online version contains supplementary material available at 10.1007/s00467-025-06779-1.

## Introduction

^99m^Technetium dimercaptosuccinic acid scan (DMSA) scintigraphy is used for functional imaging of the kidney parenchyma [1]. It provides an accurate method for assessing differential kidney function and evaluating regional parenchymal function, with a high sensitivity for detection of cortical defects in a variety of acquired and congenital kidney abnormalities [1].

Kidney scarring (KS) is permanent parenchymal damage to the kidney cortex, usually occurring post pyelonephritis (PN) [2]. Several studies have shown that DMSA scintigraphy is more accurate than other imaging modalities in detecting acute PN and subsequent cortical scarring [3]. To consider cortical abnormalities as persistent kidney scarring, the scan should be performed at least 4 to 6 months following acute infection [4–6]. Recurrent episodes of febrile urinary tract infection (UTI), delayed diagnosis of PN, and delayed initiation of antimicrobial treatment are well-known risk factors for the development of KS [7–9]. Results are inconclusive regarding the relationship between vesicoureteral reflux (VUR) and scarring, with some studies showing that only high grade VUR is a risk factor for scars [6, 10–12]. Only a subset of children with recurrent febrile UTIs will develop KS and once PN has occurred ultimate kidney scarring is independent of the presence or absence of VUR but rather on the host’s immune response [2, 11].

Congenital parenchymal kidney abnormalities are identified on DMSA scans mainly in the settings of structural kidney abnormalities or reflux nephropathy [13, 14]. DMSA scans performed in infants with VUR, without evidence of a prior UTI, showed that the common type of damage observed is diffuse inhomogeneity in tracer distribution within the kidney parenchyma with reduced differential function of the affected kidney with or without additional focal cortical defects [14–16]. It was suggested that fetal maldevelopment due to nonobstructive kidney hypodysplasia (KHD) or urinary tract obstruction is the cause for the congenital damage observed in these patients [14–18].

Kidney damage observed on DMSA scan is associated with future decreased kidney function, kidney failure, and the development of hypertension [7, 19–21]. This is especially true for patients with bilateral damage on scan [20–22]. However, these studies have not clearly defined the pattern of damage observed on DMSA scans and the distinction between peripheral focal lesions vs. diffuse inhomogeneity in parenchymal tracer distribution has not been made. The aim of our study was to assess whether differentiating between these patterns on DMSA scans can improve prediction regarding risk for recurrent UTIs and long-term kidney function.

## Material and methods

This is a retrospective study. The study was approved by the Institutional Review Board of Schneider Children’s Medical Center protocol number 0856–20-RMC. We reviewed all ^99m^Tc-DMSA scans performed at Schneider Children’s Medical center of Israel from November 2007 to February 2011. Patients with a DMSA scan performed less than 4 months post febrile UTI were excluded, to prevent the possible inclusion of acute and potentially reversible changes that can be present in acute PN. For each patient, we retrieved demographic data, information regarding episodes of febrile UTIs prior to scan, indication for scan, use of prophylactic antibiotics, results of cystography studies if conducted (the presence of VUR and its grade) and whether invasive intervention in the urinary tract prior to scan was performed and its type. Results of kidney and bladder US performed prior to the scan were recorded to examine their correlation to the scan results. US results were classified as normal, presence of hydronephrosis (without signs of KHD), other structural anomaly without signs of KHD (such as single kidney and pelvic kidney) and evidence of KHD based on kidney length for age, the presence of reduced corticomedullary differentiation or increased echogenicity [23].

**Fig. 1:**
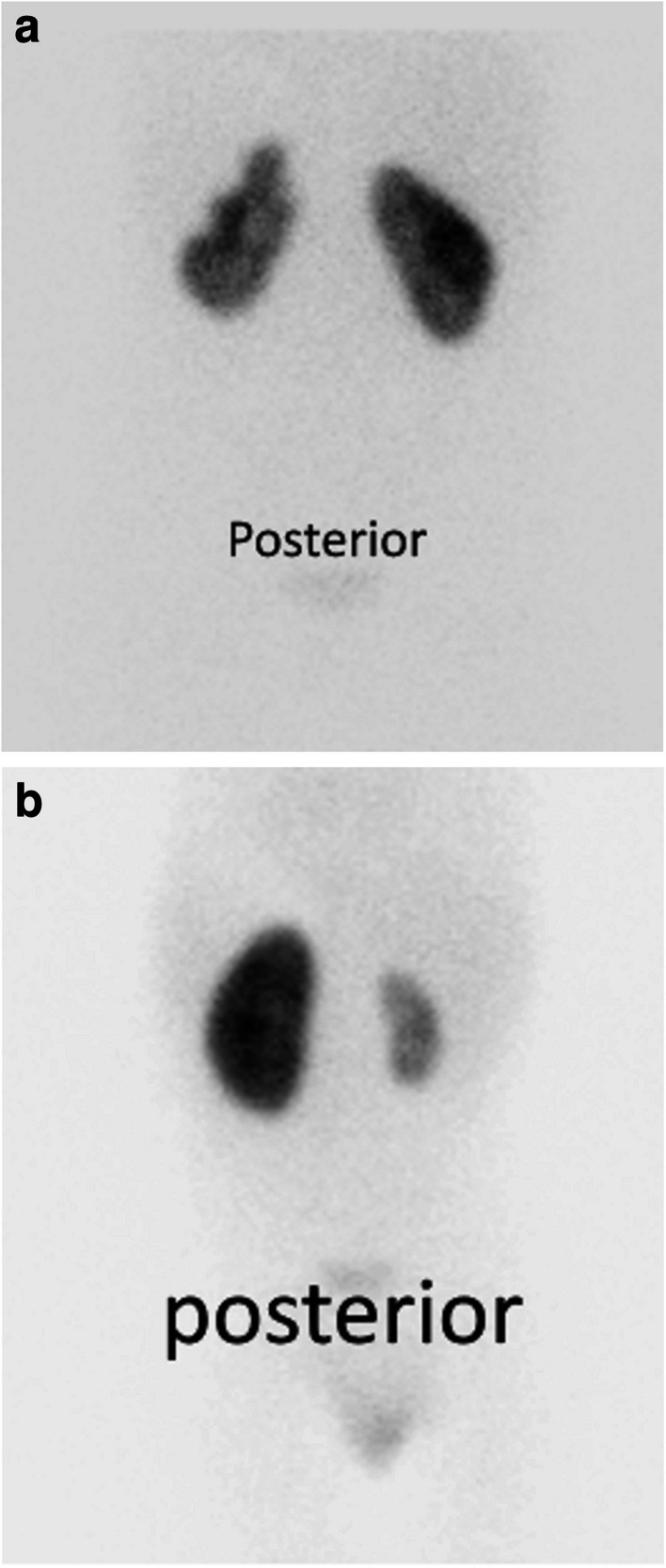
**a** Focal defects. Seven-year-old girl with recurrent febrile UTIs. Most recent infection occurred 8 months prior to the DMSA scan. A posterior view image shows wedge-shaped defects in the lateral aspect of both upper and lower poles of the left kidney. The defects distort the kidney contour and cause parenchymal volume loss. **b** Diffuse inhomogeneity. Fifteen-month-old girl with a febrile UTI 8 months prior to scintigraphy. Kidney ultrasound reported a small right kidney. A posterior view image shows a small right kidney with a functional length of 4.5 cm and a relative function of 13%. Parenchymal uptake is reduced and inhomogeneous but the renal contour is preserved

All ^99m^Tc-DMSA scans were performed at Schneider Children’s Medical Center of Israel and were analyzed by a single observer (ZBS). All scans were performed at least 4 months after the last infection and reflected permanent changes in the kidney parenchyma. Peripheral focal defects were diagnosed when wedge shaped, peripheral cortical defect with disruption of the normal contour with or without associated volume loss was identified [1]. The number of focal defects, their location and size were recorded (Fig. 1a).

Diffuse inhomogeneity was diagnosed when tracer distribution within the kidney parenchyma was reduced and inhomogeneous, but the kidney contour was preserved. These kidneys were typically smaller than the contralateral kidney with reduced functional length (Fig. 1b) [1, 14]. Regional damage was reported when the inhomogeneous parenchyma involved only a pole or part of a pole.

Outcome data were collected from patient electronic medical records and included: last recorded serum creatinine, need for surgical intervention post DMSA scan and additional episodes of UTI. Serum creatinine tests at last follow-up were measured using the same (enzymatic) method in all patients with two significant decimal digits reported. Estimated glomerular filtration rate (eGFR) was calculated using FAS (full age spectrum) equation as it enables continuity of eGFR prediction across the full age spectrum and allows for longitudinal follow-up [24]. eGFR < 75 ml/min/1.73 m^2^ by last follow-up was considered decreased kidney function and chronic kidney disease (CKD) [22].

## Statistical analysis

Descriptive statistics are presented as number and percentage for categorical variables; means and standard deviation or median and interquartile range for parametric and nonparametric variables, respectively. To compare between groups, the Pearson Chi-Square test was used for categorical parameters and analysis of variance (ANOVA) was used for continuous variables. A *p* value of < 0.05 was considered significant. Since multiple groups were compared, tests were adjusted for all pairwise comparisons using the Bonferroni correction. Logistic regression analysis was performed to look for risk factors for recurrent UTI and eGFR < 75 ml/min/1.73 m^2^. Variables found to be significant (< 0.10) on univariate analysis were included in a multivariate analysis to determine the variables most significantly associated with recurrent UTI and reduced eGFR. Linear regression was performed to identify risk factors for reduced eGFR as a continuous variable. The Wald test was used for confidence interval (CI) calculation. Statistical analysis was performed using IBM SPSS statistics, version 29.

## Results

One hundred and eight DMSA scans met inclusion criteria. Of these, three patients were excluded from further analysis, two with kidney masses and one with traumatic kidney injury. Therefore, 105 patients were included in the study cohort. Table 1 describes baseline clinical characteristics of the cohort. Of 105 patients, 48 (46%) were males. Median (IQR) age at DMSA scan was 1.9 (1.3–5) years. Sixty-five (62%) patients had at least 1 episode of febrile UTI prior to scan (range 1–9 episodes) and 40 (38%) patients did not have a febrile UTI documented prior to scan. All males in this cohort were circumcised. US studies were normal in 33 (31.4%) patients, showed hydronephrosis in 27 (25.7%), structural abnormalities in 24 (22.9%) and hypodysplasia in 21 (20%).

**Table 1:** Baseline clinical characteristics of the cohort

	*N* = 105
Age at scan, years [median (IQR)]	1.9 (1.3–5)
Males, *n* (%)	48 (46%)
fUTI prior to scan, *n* (%)	65 (62%)
US results, *n* (%)	
Normal	33 (31.4%)
Hydronephrosis	27 (25.7%)
Structural abnormalities	24 (22.9%)
Hypodysplasia	21 (20%)
Cystography prior to scan, *n* (%)	40 (38%)
No reflux	14
Reflux grade 1–2	8
Reflux grade 3	7
Reflux grade 4	5
Reflux grade 5	6
Patients on antibiotic prophylaxis, *n* (%)	39 (31.1%)
DMSA scan results, *n* (%)	
Normal	53 (50.5%)
Diffuse inhomogeneity	29 (27.6%)
Focal defects	14 (13.3%)
Diffuse inhomogeneity with focal defects	9 (8.6%)

*IQR* interquartile range, *fUTI* febrile urinary tract infection, *US* ultrasound, *DMSA* dimercaptosuccinic acid

Results of the DMSA scan were as follows: 53 (50.3%) patients had normal appearing parenchyma, and 23 (21.9%) patients had focal defects, 7 of whom with bilateral defects. Diffuse inhomogeneity was observed in 38 (36.2%) patients, 5 of them had bilateral damage, 24 had unilateral damage and 9 had segmental damage. Nine (8.6%) patients had both diffuse inhomogeneity with focal defects on scan.

## Outcome

### Recurrent UTIs

Ninety-nine patients had follow-up data at a mean period of 9.6 ± 3.26 years from inclusion. Mean age at last follow-up was 13.8 ± 5.9 years (range 2–34 years). Twenty-nine (29%) patients experienced one or more episodes of UTI during this follow-up period, and 21 (72%) of them underwent the DMSA scan after a previous UTI episode. Median number of UTI episodes was 2 (IQR: 1, 5). Risk factors for recurrent UTIs are shown in Table 2. Female sex, the number of UTI episodes prior to scan and focal defects on DMSA scan were risk factors for recurrent UTI during follow-up. On multivariate analysis the presence of focal defects on DMSA scan predicted recurrent UTIs [OR 3.89, (95% CI 1.2, 12.6), *p* = 0.024]; female sex was marginally significant [OR 3.22, (95% CI 0.995, 10.423), *p* = 0.051], but the number of UTIs prior to scan did not remain statistically significant (*p* = 0.059).

**Table 2:** Univariate analysis for recurrent UTI

	OR (95% CI)	*p* value
Female	4.9 (1.8, 13.6)	0.002
Age at 1 st UTI	1 (0.99, 1.02)	0.269
Number of UTIs prior to scan	2.2 (1.4, 3.4)	< 0.001
Diffuse inhomogeneity on DMSA scan	0.97 (0.39, 2.4)	0.94
Focal defects on DMSA scan	7.35 (2.6, 20.7)	0.0002

*UTI* urinary tract infection, *OR* odds ratio, *CI* confidence interval, *DMSA* dimercaptosuccinic acid

Looking at the four groups according to DMSA findings (Table 3) the percent of patients who developed recurrent UTIs was similar between patients with normal DMSA scan and diffuse inhomogeneity (18.3% and 22.2% respectively, *p* = 0.77), higher in patients with diffuse inhomogeneity with focal defects (44.4%) and significantly higher in patients with focal defects only (focal defects vs. normal DMSA scan 71.4% vs. 18.3%, *p* < 0.001; focal defects only vs. diffuse inhomogeneity only 71.4% vs. 22.2%, *p* = 0.006). Patients with bilateral or unilateral focal defects on DMSA scan had a significantly higher risk of recurrent UTI compared to patients without focal defects (that is, patients with normal DMSA or with diffuse inhomogeneity) (50% vs. 68.8% vs. 19.4%, respectively, *p* < 0.001). There was no difference in the rate of recurrent UTIs between patients with bilateral, unilateral, segmental or no diffuse inhomogeneity on DMSA scan (29% vs. 29% vs. 29% vs. 25%, respectively, *p* = 0.998).

**Table 3:** Long-term outcome according to findings on DMSA scan

	Normal DMSA (*n* = 49)	Diffuse inhomogeneity (*n* = 27)	Focal defects (*n* = 14)	Diffuse inhomogeneity with focal defects (*n* = 9)	*p* value
Recurrent UTI, *n* (%)	9/49 (18.3%)	6/27 (22.2%)	10/14 (71.4%)	4/9 (44.4%)	*p* < 0.001*
eGFR < 75 ml/min/1.73 m^2^, *n* (%)	1 (2%)	1 (3.7%)	0	2 (22%)	0.032**
eGFR, ml/min/1.73 m^2^ (mean ± SD)	120.1 ± 26	110.6 ± 16	106 ± 13	92 ± 34	*p* = 0.006***

*DMSA* dimercaptosuccinic acid, *UTI* urinary tract infection, *eGFR* estimated glomerular filtration rate, *SD* standard deviation

^*^Results are significant for comparison between focal defects only vs. normal DMSA scan and focal defects only vs. diffuse inhomogeneity only

^**^Results are significant for comparison between diffuse inhomogeneity with focal defects vs. normal DMSA scan

^***^Results are significant for comparison between diffuse inhomogeneity with focal defects vs. normal DMSA scan

### Long-term kidney function

Mean eGFR in the whole group at last follow-up was 112 ± 24 ml/min/1.73 m^2^. Four patients had an eGFR < 75 ml/min/1.73 m^2^ at last follow-up, one of them with a normal DMSA scan, one with diffuse inhomogeneity and two with diffuse inhomogeneity with focal defects (*p* = 0.032) (Table 3).

Looking at the four groups according to DMSA findings (Table 3), mean eGFR (± SD) was lowest in patients with diffuse inhomogeneity with focal defects compared to patients with normal DMSA scan (92 ± 34.5 vs. 120.1 ± 26.2 ml/min/1.73 m^2^ respectively, *p* = 0.006). Patients with diffuse inhomogeneity only and patients with focal defects only had a trend toward lower eGFR compared to patients with normal DMSA scan, though not reaching statistical significance (Table 3).

Patients with bilateral diffuse inhomogeneity had the lowest eGFR at last follow-up compared to patients with unilateral, segmental, or no inhomogeneity (76 ± 41 vs. 108 ± 14.5, 117 ± 17 and 117 ± 25 ml/min/1.73 m^2^, respectively, *p* < 0.03). Similarly, patients with bilateral focal defects had lower eGFR at last follow-up compared to patients without focal defects (88.9 ± 35 vs. 117 ± 23 ml/min/1.73 m^2^, respectively, *p* = 0.09). Patients with unilateral focal defects had no significant change in eGFR compared to patients without focal defects (106 ± 17 vs. 117 ± 23 ml/min/1.73 m^2^, respectively, *p* = 0.2). Linear regression analysis showed that after adjusting for sex, bilateral DMSA findings of focal defects (vs. unilateral or no defects) and bilateral diffuse inhomogeneity (vs. unilateral or no inhomogeneity) were associated with reduced eGFR at last follow-up: [focal defects: *r* = − 0.34 (95% CI − 21.4, − 6.1), *p* < 0.01; diffuse inhomogeneity: *r* = − 0.26 (95% CI − 18.7, − 3.3), *p* = 0.006].

## Discussion

DMSA scan is a sensitive tool to diagnose abnormalities of kidney parenchyma. The presence of kidney damage on DMSA scan has been shown to correlate with decreased kidney function on long-term follow-up in several studies [20, 21]. Damage observed on DMSA scan can either be congenital (for example, due to kidney dysplasia or obstruction) or acquired due to post pyelonephritic scars [1, 14]. Our hypothesis is that focal defects most probably represent acquired KS post pyelonephritis while diffuse inhomogeneity likely represents congenital maldevelopment of kidney parenchyma [11, 14]. The aim of our study was to differentiate between these different patterns observed on DMSA scan and examine their effect on future episodes of UTI and long-term kidney function.

We show that in addition to female sex, patients with focal defects (but not those with diffuse inhomogeneity) exhibited an independent risk for UTI recurrence. Analysis of the RIVUR and CUTIE cohorts showed that kidney scarring at baseline, though rare, was also found to be a risk factor for recurrent UTIs [25]. In these cohorts, however, baseline DMSA scan was performed within 8 weeks of infection likely diagnosing acute PN and not permanent damage. In addition, they did not differentiate between focal vs. diffuse inhomogeneity as kidney scarring was defined as focal or diffuse decreased uptake of DMSA, with and without loss of contours or cortical thinning with decreased volume. Our study shows that while focal defects appear to be a significant risk factor for recurrent UTI, diffuse inhomogeneity is not: the prevalence of recurrent UTI in patients with diffuse inhomogeneity was similar to that of patients with normal DMSA scan, while patients with focal defects only or patients with diffuse inhomogeneity with focal defects had a much higher prevalence of recurrent UTI. Our results are like those described by Loukogeorgakis et al. who showed that in patients with VUR the risk for breakthrough UTI is higher in patients with kidney scarring but not in patients with reduced differential kidney function [26].

The interrelationship between VUR and recurrent UTI on KS formation has been studied extensively [11, 25, 27]. Even though high-grade VUR increases the risk of recurrent UTIs, a high percentage of patients with high grade VUR will have a normal DMSA scan [11]. In addition, less than 40% of children with recurrent UTIs have VUR [28]. It has been suggested that genetic polymorphisms which alter the expression of innate immunity increase susceptibility for recurrent UTIs [11, 29]. Our results show that focal defects, most probably representing KS, both unilateral and bilateral, are strongly correlated with recurrent UTIs, as they may reflect the host’s capability to respond to invading uropathogens. Beetz et al. showed that a high percentage of female patients experience recurrent symptomatic UTIs even after successful surgical correction, emphasizing the presence of additional risk factors for recurrent UTIs other than reflux [30].

Regarding long-term kidney function, we show that both bilateral focal defects and diffuse inhomogeneity on DMSA scan are mildly predictive of reduced eGFR during follow-up. In this study, mean eGFR was above 90 ml/min/1.73 m^2^ in the four groups of patients according to DMSA results. However, patients with diffuse irregularity with focal defects had a significantly lower eGFR at last follow-up compared to patients with normal DMSA scan, with 2 (22%) patients in this subgroup developing an eGFR < 75 ml/min/1.73 m^2^ at last follow-up, suggesting established CKD. This indicates that scan results may help us identify (in cases not obviously diagnosed by kidney function dynamics and kidney US) the subset of patients with the highest risk for kidney deterioration, mainly those with likely both congenital maldevelopment of kidney parenchyma and scars on scan.

Like previous studies, this study also shows that patients with bilateral findings on DMSA scan also have a significantly lower eGFR at follow-up compared to patients with normal DMSA scan. Geback et al. and Wennerstrom et al. showed on long-term follow-up of more than 15 years, that patients with bilateral damage on DMSA scan had lower eGFR compared to patients with unilateral or no damage on scan [20, 31].

Kidney sonography is not sensitive enough for the diagnosis of KS [13, 32]. Results of this study suggest that performing a DMSA scan in patients with suspected kidney hypodysplasia by US after an episode of febrile UTI, or in patients with recurrent febrile UTIs may help identify patients with diffuse inhomogeneity and focal defects and patients with bilateral KS, who have the highest risk for recurrent UTI and kidney deterioration.

This study has a few limitations, mainly its retrospective design and the small sample size. In addition, the follow-up period was limited. It is possible that a longer follow-up period could have shown a more accurate image of the impact of kidney scaring on kidney function. VCUG was not performed in all patients prior to scan. Therefore, the presence of VUR was not assessed in all patients and could not be included to the logistic regression model. Another limitation is that DMSA scans were reviewed by one expert only; therefore, an inter-observer variability of DMSA results is possible.

Our hypotheses that focal defects most likely represent acquired scarring while diffuse inhomogeneity represents congenital damage requires further validation since an overlap can exist between these patterns of congenital and acquired lesions on DMSA scans. Therefore, a clear differentiation regarding etiology cannot be made based on DMSA alone [17, 18]. Nevertheless, our results that patients with focal defects have an increased risk of recurrent UTs and that patients with diffuse inhomogeneity with focal defects have the highest risk of developing CKD suggest that significant predictions can be made based on this imaging modality. Future prospective studies that distinguish between patterns of damage observed on DMSA are required to determine whether this distinction has implications regarding clinical management and long-term outcome.

## Supplementary Information

Below is the link to the electronic supplementary material.

Graphical abstract (PPTX 78.1 KB)

## Data Availability

The datasets generated during and/or analyzed during the current study are available from the corresponding author on reasonable request.
