# Supplementary material for: Patterns of damage observed on dimercaptosuccinic acid kidney scans and future risk of urinary tract infections or reduced kidney function
Source: Pediatr Nephrol. 2025 Apr 28;40(9):2863–9. doi: 10.1007/s00467-025-06779-1 (PMC12296764; doi:10.1007/s00467-025-06779-1)
Supplement: Supplementary file 1 — Graphical abstract (PPTX 78.1 KB) [file 467_2025_6779_MOESM1_ESM.pptx]

## Slide 1
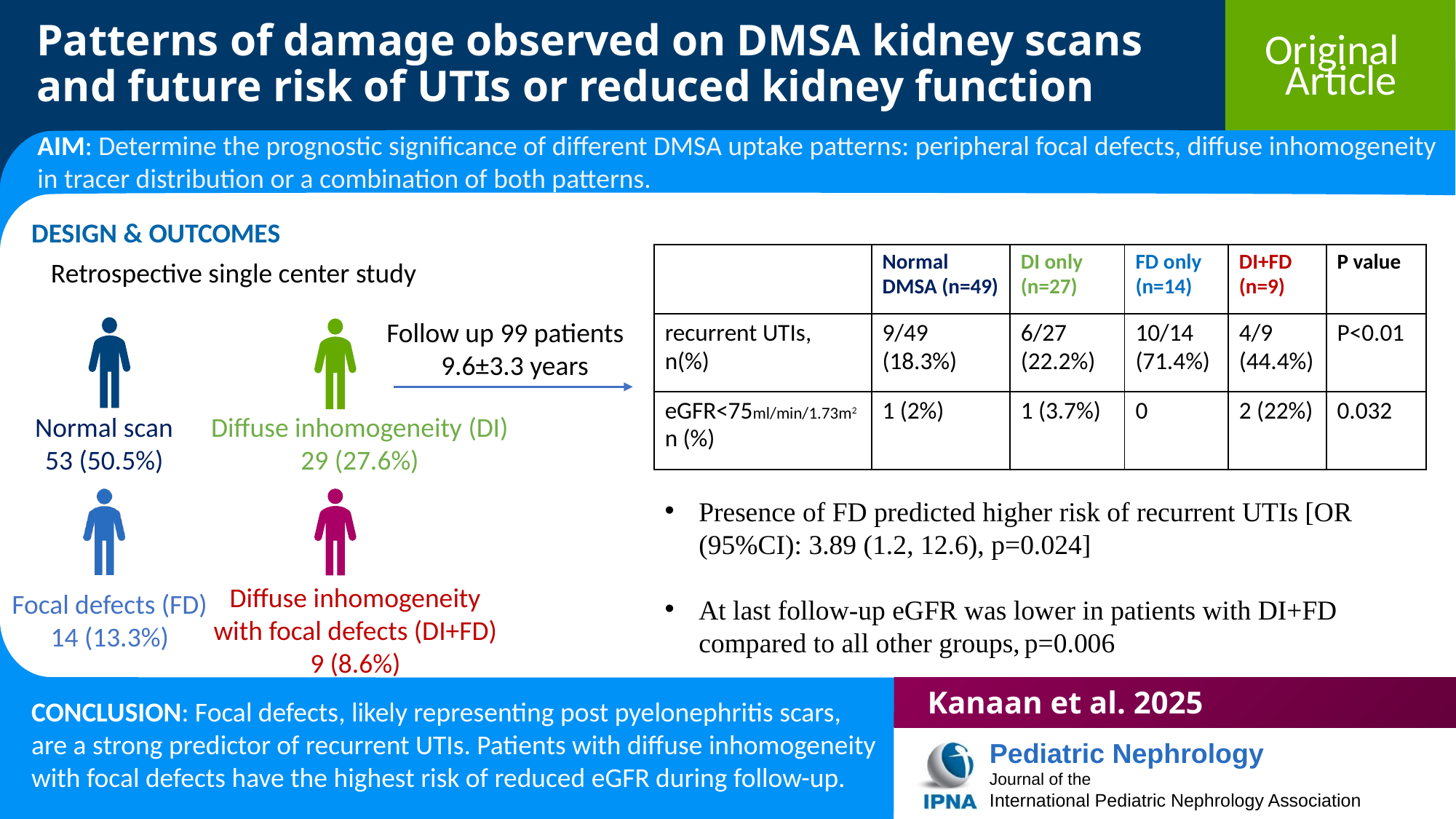

Patterns of damage observed on DMSA kidney scans and future risk of UTIs or reduced kidney function
AIM: Determine the prognostic significance of different DMSA uptake patterns: peripheral focal defects, diffuse inhomogeneity in tracer distribution or a combination of both patterns.
DESIGN & OUTCOMES
| | Normal DMSA (n=49) | DI only (n=27) | FD only (n=14) | DI+FD (n=9) | P value |
| --- | --- | --- | --- | --- | --- |
| recurrent UTIs, n(%) | 9/49 (18.3%) | 6/27 (22.2%) | 10/14 (71.4%) | 4/9 (44.4%) | P<0.01 |
| eGFR<75ml/min/1.73m2 n (%) | 1 (2%) | 1 (3.7%) | 0 | 2 (22%) | 0.032 |
Retrospective single center study
Follow up 99 patients
9.6±3.3 years
Normal scan 53 (50.5%)
Diffuse inhomogeneity (DI)
29 (27.6%)
Presence of FD predicted higher risk of recurrent UTIs [OR (95%CI): 3.89 (1.2, 12.6), p=0.024]
At last follow-up eGFR was lower in patients with DI+FD compared to all other groups, p=0.006
Diffuse inhomogeneity with focal defects (DI+FD)
9 (8.6%)
Focal defects (FD)
14 (13.3%)
Kanaan et al. 2025
CONCLUSION: Focal defects, likely representing post pyelonephritis scars, are a strong predictor of recurrent UTIs. Patients with diffuse inhomogeneity with focal defects have the highest risk of reduced eGFR during follow-up.
